# Risk factors and biomarkers of life-threatening cancers

**DOI:** 10.3332/ecancer.2015.596

**Published:** 2015-11-24

**Authors:** Philippe Autier

**Affiliations:** University of Strathclyde Institute of Global Public Health at iPRI, International Prevention Research Institute (iPRI, www.i-pri.org), Espace Européen d’Ecully, Bâtiment G, Allée Claude Debussy, Ecully ouest Lyon 69130, France

**Keywords:** biomarker, cancer, chemoprevention, phenotype, risk factor, screening

## Abstract

There is growing evidence that risk factors for cancer occurrence and for cancer death are not necessarily the same. Knowledge of cancer aggressiveness risk factors (CARF) may help in identifying subjects at high risk of developing a potentially deadly cancer (and not just any cancer). The availability of CARFs may have positive consequences for health policies, medical practice, and the search for biomarkers. For instance, cancer chemoprevention and cancer screening of subjects with CARFs would probably be more ethical and cost-effective than recommending chemoprevention and screening to entire segments of the population. Also, the harmful consequences of chemoprevention and of screening would be reduced while effectiveness would be optimised. We present examples of CARF already in use (e.g. mutations of the breast cancer (BRCA) gene), of promising avenues for the discovery of biomarkers thanks to the investigation of CARFs (e.g. breast radiological density and systemic inflammation), and of biomarkers commonly used that are not real CARFs (e.g. certain mammography images, prostate-specific antigen (PSA) concentration, nevus number).

## Introduction

The environmental, lifestyle, and genetic causes of cancer have received considerable attention over the past five decades. Hundreds of studies have unveiled some of the risks factors involved in the occurrence of cancer. By comparison, few studies have examined risk factors for being diagnosed with a life-threatening cancer versus being diagnosed with an indolent cancer that would never be life-threatening. In other words, what are the characteristics of subjects likely to be diagnosed with a ‘pussy cat’ cancer (i.e. non-life-threatening or easily curable) or with a ‘tiger’ cancer (i.e. deadly in the absence of an efficient treatment)? Do subjects diagnosed with a metastatic cancer present characteristics that distinguish them from subjects without cancer or from subjects with early-stage cancer, a part from attending screening? These characteristics may be linked to a variety of personal attributes, hereditary traits, lifestyle factors and exposures to substances or circumstances associated with a greater likelihood developing of a ‘tiger’ rather than of a ‘pussy cat’ cancer.

Cancers may be life-threatening either because they are diagnosed at a late stage, when metastases have spread into the lymph nodes or in distant organs, or because the cancer has an aggressive phenotype. Indeed, it is well documented that the more aggressive a cancer is, the greater the likelihood it will be diagnosed when at a late stage. However, a sizeable proportion of subjects dying from cancer are diagnosed with a cancer that is apparently still localised. Conversely, a fraction of subjects with advanced cancer survive for a long time even though they received the same amount of attention as other patients with the same stage of cancer.

Recent epidemiological and clinical studies have documented that risk factors for cancer occurrence and for cancer death are not necessarily the same, and that pre-diagnostic, non-tumour-related risk factors could be different for slow progressing or for aggressive cancers [1, 2]. Identification of subjects at higher risk to develop a potentially deadly cancer could thus be based on the recognition of specific personal genetic, lifestyle, or environmental characteristics, or on the measurement of non-tumour biomarkers, all factors that we term ‘cancer aggressiveness risk factor’ (CARF) hereafter.

## Risk factors for cancer occurrence and for cancer death

Cancers affecting the same organ are no longer considered as a single disease entity. The wisdom that risk factors for cancer occurrence would be the same as for the risk for aggressive cancer or of cancer death is not correct. For instance, reproductive factors have a strong influence on the risk of breast cancer but little influence on the risk of breast cancer death [2]. Adiposity is associated with reduced breast cancer risk in premenopausal women. However, the risk of death from breast cancer in premenopausal women increases with adiposity [3]. High fertility is associated with reduced risk of breast cancer. However, women giving birth in their 40s have become increasingly common, and breast cancer occurring in the first 2 years after childbirth is known to be more lethal [4]. Another example is smoking in prostate cancer. If smoking is not a risk factor for prostate cancer occurrence, it seems to be associated with the occurrence of fatal prostate cancer [5].

The search for hereditary, lifestyle, and environmental factors that would be involved in the occurrence of potentially life-threatening cancers has really started only after 2000, mainly because of the longstanding false impression that risk factors for cancer occurrence and for cancer death were similar. In 1990, an International Agency for Research on Cancer (IARC, Lyon, France) Scientific Publication on the causes, occurrence, and control of cancer stated that ‘the fact of death from cancer is rarely of interest in epidemiology’ [6]. There was also the belief from the pre-screening era that any lesion that is labelled as ‘cancer’ by histological examination would necessarily be life-threatening.

## CARF, health policies, and patient management

The availability of CARFs allowing risk stratification would have several implications for health policies and medical practice (Table 1).

Firstly, primary prevention efforts could concentrate on subjects with CARF(s). Personalised counselling based on the presence of CARF could be more effective than primary prevention campaigns spread over entire populations.

Secondly, because the principal goal of cancer screening is the prevention of cancer death through the detection of cancers at an early, curable stage, CARF could allow the prioritisation of screening efforts towards subjects with CARF and avoid screening in subjects having a low probability to develop an aggressive cancer. CARF could boost the cost-effectiveness of screening because it could be directed to subjects at higher risk of cancer death and not just to subjects at higher risk of cancer. Moreover, if an efficient screening method exists, knowledge by subjects that they harbour a CARF might motivate them to participate in screening.

Third, subjects diagnosed with an apparently good prognosis cancer but who harbour a CARF could be at a higher risk of relapse, which could lead to recommending more intense management of these subjects. Using this logic, availability of CARF may help in selecting subjects with early stage cancer for inclusion in randomised trials for testing the efficacy of adjuvant therapy. CARF may provide useful information for the selection of patients with *in situ*, borderline or early stage cancer for active surveillance (e.g. if no CARF is present) or for immediate treatment (e.g. if CARF is present). For instance, a history of pregnancy in the two years preceding a diagnosis of breast cancer or being diabetic when breast cancer is found are two CARFs associated with poorer prognosis, even when the tumour has been detected at an early stage (e.g. size less than 20 mm and oestrogenic receptor positive) [4, 7, 8].

Fourth, the discovery of CARF can have an invaluable role in research on biological mechanisms involved in the occurrence of deadly cancers.

## When would CARF be most relevant?

The availability of CARF may help to identify subjects with high or low probability of being diagnosed with an aggressive cancer.

Availability of CARF would be mostly valuable when the incidence of a cancer is much greater (say more than two times greater) than the mortality due to that cancer, meaning that the majority of cancers would not be a cause of death. Over the last 30 years, screening and the availability of imaging and biopsy methods allowing the detection of steadily smaller tumours has led to considerable increases in the incidence to mortality ratio because of over diagnosis. Over diagnosis is the detection of a lesion that has all the features of a cancer under the microscope but does not progress and will thus never be life-threatening. These cancers that do not clinically behave like cancers are pseudocancers whose frequency has been boosted by the advent of screening. Over diagnosis is common in breast, prostate, and thyroid cancer, and in cutaneous melanoma. Over diagnosis is known to be an issue for computerised tomography (CT) scan lung cancer screening. Over diagnosis also encompasses the detection of *in situ* or of borderline cancers, most of which will never transform into an invasive cancer or be life-threatening. For instance, before the mammography screening era, *in situ* breast cancers represented less than 5% of all breast cancers. In areas where mammography screening is widespread, 15 to 20% of breast cancers are *in situ*. Although we still have poor knowledge of the natural evolution of untreated *in situ* breast cancer [9], these lesions are nearly always treated. Treatment may be aggressive, with mastectomy (sometimes bilateral), radiotherapy, adjuvant chemotherapy and search for lymph node metastases despite that the lack of evidence on the most adequate management of these tumours. Because the finding of *in situ* breast cancer generally entails treatment, the mammography detection of these lesions is usually considered as over diagnosis [10].

Using this logic, focusing screening efforts on subjects with CARF is likely to increase the cost-effectiveness of screening and lower the harms due to screening. CARF may also assist in the prioritisation of referral to diagnosis and specialised care.

## The quest for CARF

Generally speaking, studies done so far have not identified many CARFs that could be proposed for the risk stratification of subjects. However, several CARFs are already well documented and some promising avenues for research have been identified. We outline some examples here later.

### Hereditary factors

Subjects with germline mutation associated with increased risk of cancer may also be at increased risk of aggressive cancer. The best known example are mutations of the BRCA gene that confers a higher lifetime risk of breast cancer, and breast cancers found in BRCA women tend to be more aggressive, with greater frequency of the triple negative phenotype [11].

### Screen detectability of cancer

In the 1980s and 1990s, it was thought that screen-detected cancers would display signatures of their aggressiveness. For instance, images suggesting large cancers on mammograms or high serum concentration of biomarkers (e.g. the PSA) would reflect the presence of more aggressive cancer. However, if screen-detected cancer can indeed be fatal, these cancers are on average less aggressive and have a better prognosis that is independent of risk factors known to predict cancer survival. Conversely, cancers that were missed by screening or developed in the interval between two screening rounds (i.e. the interval cancers) are more aggressive and have a worse prognosis than screen-detected cancers. Large suspicious mammography images containing calcium deposits often indicate the presence of *in situ* cancer that is seldom life-threatening.

In the 1990s, small size, single institution studies suggested that the greater the increase in PSA level between two screening rounds, the more likely the presence of an aggressive prostate cancer (i.e. Gleason Score of 7 or more). It turned out that the reverse was true. For instance, a recent re-analysis of the prostate, lung, colorectal, and ovarian (PLCO) trial showed that the magnitude of changes in PSA level between screening rounds is not a predictor of cancer aggressiveness (Table 2).

Hence, for two of the most common cancers, currently available screening methods are not optimal in their ability to early detect the most life-threatening cancers, and future research needs to test the capacity of new screening methods to specifically detect potentially deadly cancers when still at a curable stage.

### Radiological density of breasts

The risk of breast cancer is four to five times greater in women with radiographically dense breasts than in women with little or no density in the breast [12, 13]. Breast radiological density is an attractive CARF for several reasons. Cancer detection is less sensitive in radiographically dense breasts, and thus, interval cancers tend to be more common in women with dense breast [14–16]. Cancers found in dense breasts have a more aggressive phenotype and are more advanced than when found in fatty, non-dense breasts [12, 17, 18]. Hence, there is growing support for informing women with dense breasts about their higher risk of breast cancer and greater probability of false-negative mammographic examination. There is, however, no firm evidence that other breast examinations (e.g. ultrasonography) would improve the ability of screening to detect mammographically silent cancers that could be life-threatening. In any case, for legal and financial reasons, a growing number of states in the USA requires that radiologists notify women about the radiological density of their breasts (http://www.diagnosticimaging.com/breast-imaging/breast-density-notification-laws-state-interactive-map).

### Obesity, diabetes, and low-grade systemic inflammation

Cancers diagnosed among obese and diabetic subjects have a poorer prognosis, because they tend to be of more aggressive phenotype, are more advanced at diagnosis and are less sensitive to treatments [3, 19–25]. More advanced stages would not just be due to a greater difficulty to detect cancer in obese subjects or to lower propensity of these subjects who participate to screening. Mechanisms by which adiposity and glucose metabolism disorders would influence cancer phenotype are not well known. The low-grade inflammation that prevails in most obese and diabetic subjects could be the factor underlying the occurrence of more aggressive cancer.

Indirect evidence for a link between systemic inflammation and aggressive cancer phenotype is provided by prospective studies on vitamin D concentration and the risk of subsequent cancer. Subjects with systemic inflammation have lower vitamin D levels [26], and low vitamin D levels are associated with more aggressive and more advanced breast, colorectal, and prostate cancer, and of cutaneous melanoma [27–31].

Studies like those on the toll-like receptor 4 [25, 32] represent significant strides in the understanding between inflammatory processes and cancer. It is hoped that the vast body of data accumulating on inflammation and cancer will end up in the discovery of biomarkers allowing the identification of subjects at high risk of aggressive cancer and in the discovery of new cancer treatment modalities based on the control of inflammatory processes.

### Nevus count and cutaneous melanoma

The number and size of skin nevi is the strongest predictor of one’s chance of being diagnosed with a melanoma. Skin self-surveillance and skin screening are usually recommended to subjects with numerous nevi. The incidence of melanoma has dramatically increased over the last four decades, in part because of greater exposure to ultraviolet radiation during holidays [33], and in part because of steadily increasing rates of nevus excision [34]. The expected reductions in the risk of melanoma death associated with the screening of subjects with numerous nevi rests on the caveat that these subjects would be at higher risk of melanoma death than subjects with few nevi. However, the few available data do not suggest a higher risk of melanoma death associated with increased number of nevi [35]. A high mitotic rate of melanoma cells is associated with a poorer prognosis. A recent study found no association between nevus count and the mitotic rate [36]. Hence, there is no evidence so far that a high nevus count could represent a CARF for melanoma and preferential screening of subjects with numerous nevi is probably no more effective than screening subjects with few nevi.

## Search for CARF in the context of cancer screening

Subjects participating in screening are generally diagnosed with smaller and earlier-stage cancer than subjects not participating to screening. Cancer earliness may be due to the detection of cancers that would have been more advanced and more life-threatening if they had been symptomatic (i.e. lead time cancers). Cancer earliness may also be due to the detection of pseudocancers that would have never been symptomatic (i.e. length time cancers). Hence, a greater proportion of early-stage cancer in screened rather than in unscreened subjects may be linked to screening efficiency (ability to detect cancer at an early, asymptomatic stage) and to screening-induced over diagnosis. In addition, screen-detected cancers are less aggressive, while interval cancers are more aggressive. Finally, subjects not participating in screening are those who, in the absence of screening, would be at highest risk of being diagnosed with an advanced cancer and to die from it.

Because of the complex relationships between screening and cancer phenotype, the search for CARF must be aware of possible biases that could be introduced by the screening history of subjects.

## Ethics

Targeted screening on the basis of CARF would probably be more ethical than recommending screening to entire segments of the population that are just defined by age and sex. The harmful consequences of screening would be reduced, while effectiveness would be optimised.

A key issue for the screening of breast, prostate, and lung cancer is that subjects whose lives are saved by screening are not necessarily the same subjects that incur the harmful consequences of screening. For instance, it was estimated for the United Kingdom that for one death prevented thanks to mammography screening, there are three women with over diagnosed cancer [10]. Because in the United Kingdom, mammography screening is proposed every three years, the ratio of three over diagnosed cancers to one life saved must be higher in most other countries where mammography screening is done every year or every two years. Hence, if a CARF allowed the risk stratification of women for their likelihood to have an indolent or an aggressive breast cancer, screening could be concentrated in women at high risk to have a potentially deadly breast cancer and probably abandoned in women at low risk of such cancer.

## Conclusion

The possibility of identifying subjects at high or at low risk of developing a potentially deadly cancer may represent a new frontier in cancer research that would have many implications for screening policies, chemoprevention, and decision on treatment options for early-stage cancer which could integrate a better evaluation of the risk of relapse and the aspiration to avoid overtreatment.

## Conflicts of interest

The authors declare that there is no conflicts of interest.

## Figures and Tables

**Table 1.** Examples of domains of application of cancer aggressiveness risk factors (CARF).

**Primary prevention** Definition of population subgroups most likely to benefit from primary prevention policies Personalised prevention according to CARF presented by subjects
**Screening** Definition of population subgroups in which the balance between harms and benefits would be optimal Personalised screening (age at start, method(s) of choice, intensity) according to CARFs presented by subjects Identification of subjects with in benign lesion (e.g. breast hyperplasia) to develop a life-threatening invasive cancer Identification of subjects with in situ or borderline cancers most likely to develop a life-threatening invasive cancer Characterisation of subjects participating to screening most likely to develop and interval cancer
**Patient management** Selection of patients with *in situ***,** borderline and early-stage cancer for active surveillance (no CARF) rather than immediate treatment (CARF is present) More intense follow-up of patients with apparently good prognosis cancer if early detection of relapse increases survival Selection of subjects with apparently good prognosis cancer for the evaluation of adjuvant therapies
**Basic research** Knowledge of biological mechanisms involved in CARFs may lead to the discovery of: biomarkers with better sensitivity and specificity for indentifying subjects at higher rish of potentially deadly cancer new prevention, screening, and treatment modalities

**Table 2.** Risk of aggressive prostate cancer (Gleason score ≥ 7) by PSA variation over time (after Boniol *et al*, 2015).

PSA variation	Subjects	Prostate cancers	Gleason score ≥ 7	OR^*^	95%CI	
					Lower bound	Upper bound
< -50%	872	25	16	0.75	0.33	1.71
-50%; -20%	5199	263	149	1.01	0.77	1.32
-20%; +20%	15723	1424	806	1.00	**Reference**	
+20%; +50%	5697	659	371	0.98	0.82	1.19
> +50%	3766	381	214	0.95	0.75	1.19

* Model adjusted for age and baseline PSA value.
